# A pyroptosis-related gene signature for prognosis and immune microenvironment of pancreatic cancer

**DOI:** 10.3389/fgene.2022.817919

**Published:** 2022-08-29

**Authors:** Sifan Tao, Li Tian, Xiaoyan Wang, Yajun Shou

**Affiliations:** ^1^ Department of Gastroenterology, The Second Xiangya Hospital, Central South University, Changsha, Hunan, China; ^2^ Department of Gastroenterology, The Third Xiangya Hospital, Central South University, Changsha, Hunan, China; ^3^ Research Center of Digestive Disease, Central South University, Changsha, Hunan, China

**Keywords:** pyroptosis, pancreatic cancer, immune microenvironment, prognostic model, therapeutic response prediction

## Abstract

Pancreatic cancer is one of the most lethal tumors owing to its unspecific symptoms during the early stage and multiple treatment resistances. Pyroptosis, a newly discovered gasdermin-mediated cell death, facilitates anti- or pro-tumor effects in a variety of cancers, whereas the impact of pyroptosis in pancreatic cancer remains unclear. Therefore, we downloaded RNA expression and clinic data from the TCGA-PAAD cohort and were surprised to find that most pyroptosis-related genes (PRGs) are not only overexpressed in tumor tissue but also strongly associated with overall survival. For their remarkable prognostic value, cox regression analysis and lasso regression were used to establish a five-gene signature. All patients were divided into low- and high-risk groups based on the media value of the risk score, and we discovered that low-risk patients had better outcomes in both the testing and validation cohorts using time receiver operating characteristic (ROC), nomograms, survival, and decision analysis. More importantly, a higher somatic mutation burden and less immune cell infiltration were found in the high-risk group. Following that, we predicted tumor response to chemotherapy and immunotherapy in both low- and high-risk groups, which suggests patients with low risk were more likely to respond to both immunotherapy and chemotherapy. To summarize, our study established an effective model that can help clinicians better predict patients’ drug responses and outcomes, and we also present basic evidence for future pyroptosis related studies in pancreatic cancer.

## Introduction

Pancreatic cancer (PAAD), which is primarily composed of pancreatic ductal adenocarcinoma, is one of the most fatal malignancies in the United States, with a survival rate of about 10% (Siegel et al., 2021). The poor prognosis and stable incidence rates of PAAD cases were not only associated with increased exposure to risk factors such as obesity, diabetes, tobacco use, and alcohol consumption, but also with nonspecific symptoms at the early stage (Stolzenberg-Solomon et al., 2013; Rebours et al., 2015; Walter et al., 2016). Worse still, only modest progress has been achieved in reducing the mortality rate of PAAD. Though immunotherapy has proved to be a promising treatment in many other malignancies, few PAAD patients benefited from ICIs (Torphy et al., 2018; Galluzzi et al., 2020). The “cold” tumor microenvironment is one of the primary reasons for its immunotherapy resistance (O’Donnell et al., 2019). The tumor microenvironment of PAAD is mainly composed of immunosuppressive cells, such as tumor-associated macrophages, myeloid-derived suppressor cells, and regular T-cells (Clark et al., 2007). Additionally, it is believed that an unusually intense desmoplastic reaction surrounding PAAD contributes to the formation of a barrier that prevents immune infiltration and chemotherapy exposure (Provenzano et al., 2012; Ho et al., 2020). Therefore, it is critical to investigate the molecular pathways related to PAAD microenvironment.

Pyroptosis is defined as the caspase (CASP) family-driven programmed necrotic cell death mediated by gasdermin (GSDM) (Shi et al., 2015). When triggered by bacterial, viral, toxin, or chemotherapy, pyroptosis can release pro-inflammatory cytokines and immunogenic material, promoting the activation and infiltration of immune cells (Loveless et al., 2021; Yu et al., 2021). Pyroptotic cell death is characterized by cellular swelling and bubble-like protrusions forming on the cell membrane surface, as well as the release of IL1 and IL18 (Loveless et al., 2021; Yu et al., 2021). Cancers of all forms are closely related to pyroptosis (Yu et al., 2021). On one hand, inducing pyroptosis was originally considered a promising therapeutic strategy for increasing anti-tumor immune response. On the other hand, the activation of multiple signaling pathways and the release of cytokines can lead to tumorigenesis and drug resistance (Xia et al., 2019). The connection between PAAD and pyroptosis is still unclear. Recent work demonstrated that STE20-like kinase 1 slowed PAAD progression by triggering ROS-mediated pyroptosis, implying that pyroptosis may be a potential therapeutic target for PAAD (Cui et al., 2019).

One possible reason for the depressing outcomes of immunotherapy is that PAAD cells can avoid cell death induction (Chen et al., 2021). Thus, we sought to advance our understanding of the pyroptotic pathway in PAAD and construct a pyroptosis-related gene (PRG) prognostic signature. Our study provided an effective prognostic model as well as basic evidence for subsequent pyroptosis-related studies in PAAD.

## Materials and methods

### Data extraction

**FIGURE 1:**
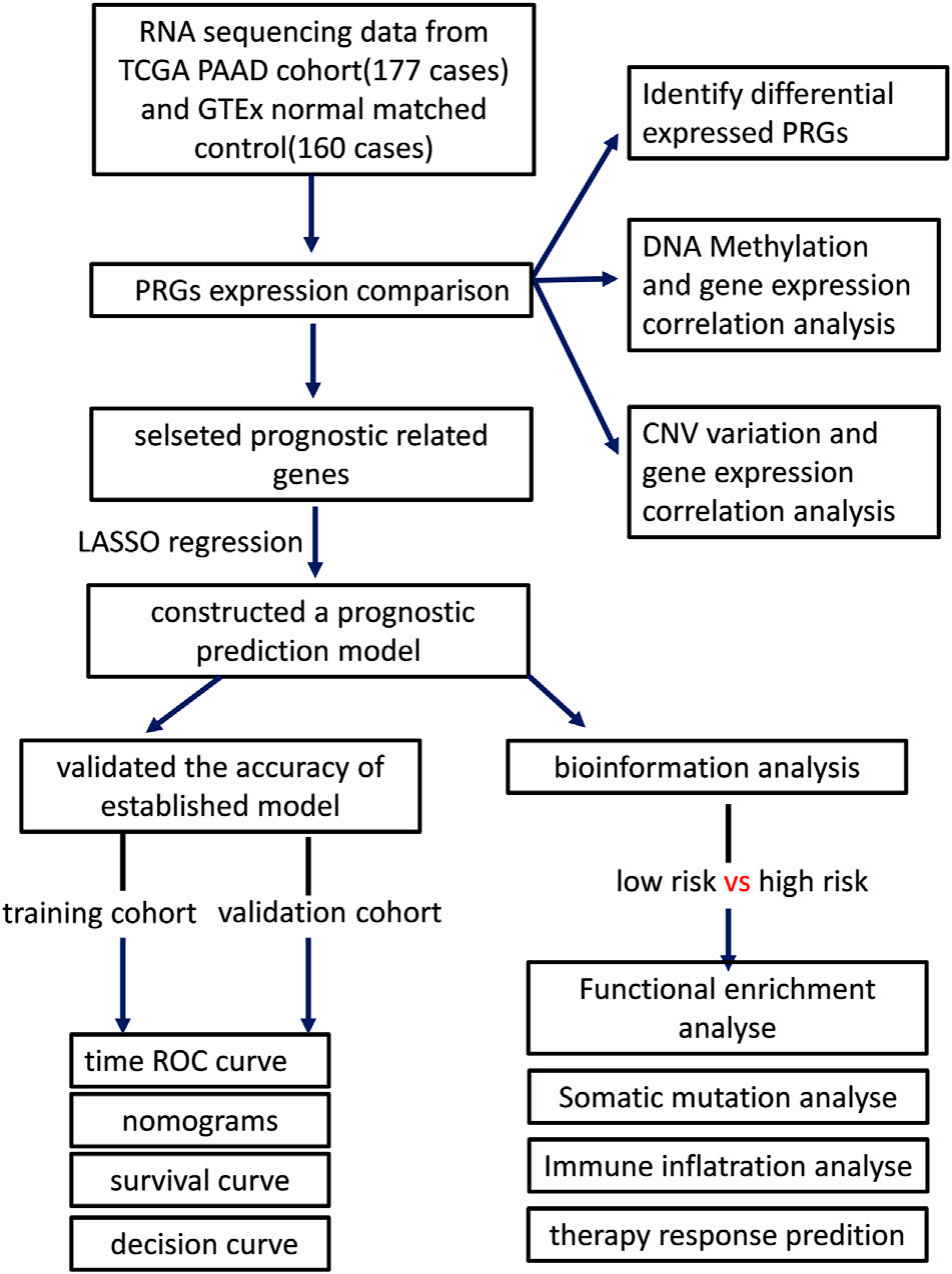
The workflow of our study.

The workflow of our study is revealed in Figure 1. The UCSC Xena (Goldman et al., 2020) (Xean, http://xena.ucsc.edu/) was used to obtain the RNA sequencing profile and clinical following data of the TCGA-PAAD cohort and GTEx cohort. Xena was also implemented to integrate normalized counts from TCGA-PAAD and GTex cohort due to limited matched controls in the TCGA-PAAD cohort. All PAAD patients without survival following were excluded in this study. In this cohort, there are 177 PAAD patients and 167 normal pancreatic tissue. The GISTIC copy number dataset and DNA methylation data for all selected patients were obtained from cBioportal (https://www.cbioportal.org/), while the somatic mutation data of patients was downloaded from TCGA (https://portal.gdc.cancer.gov/). Additionally, we downloaded two extra GEO datasets (GSE28735 and GSE62452, https://www.ncbi.nlm.nih.gov/geo/) and ICGC sequencing profiles from ICGC (https://daco.icgc.org/) as independent validation cohorts (Zhang et al., 2012; Yang et al., 2016).

### Identify differential expressed genes and perform functional analysis

The 33 PRGs were selected from a previously published study and are listed in Supplementary Table S1 (Ye et al., 2021). The “DESeq2” package was used to identify differentially expressed genes (DEGs) (Love et al., 2014). Additionally, we conducted correlation analyses of gene expression and methylation using the cBioportal (http://cbioportal.org) (Cerami et al., 2012). The Mann-Whitney or unpaired *t*-test was used to investigate gene expression differences across distinct copy number variations (CNV). The function of DEGs was analyzed using KEGG enrichment analysis and gene set enrichment analysis (GSEA) *via* the “clusterProfiler” R package (Yu et al., 2012). *p*-values < 0.05 were defined as statistically significant.

### The construction of prognostic prediction models

To begin, univariate cox regressions were utilized to examine the relationships between individual 33 PRGs and overall survival (OS) in the TCGA cohort. *p*-value < 0.05 was set as the threshold to identify prognostic-related PRGs. LASSO regression analysis was then used to select significant PRGs and minimize the likelihood of overfitting. Based on these selected PRGs, the prognostic model was constructed using multivariate cox regression analysis. The risk score for OS was constructed as the following formula:

$$risk\;score=\sum_{i}^{5}Xi*\beta i$$

Where *X* represents the gene expression level and *β* represents the regression coefficient calculated by multivariate Cox regression. All patients were separated into high- and low-risk groups based on the media value of the risk score.

### Validation of the prognostic prediction model

To evaluate the accuracy of the prediction model, time receiver operating characteristic (ROC) curve, nomograms, Kaplan-Meier survival curve, and decision curve were established in the TCGA cohort and validation ICGC cohort. The ROC curves at 1-, 3-, and 5- years were generated using the R package “timeROC” (Blanche et al., 2013). The Kaplan-Meier survival curve was generated by using the R package “survival” (Grambsch, 2000). The decision curve and the following clinic impact curve were finished by the R package “rmda” (Brown, 2018). And the R package “regplot” (Marshall, 2020) was used to perform the nomogram analysis.

### Molecular variation analysis and tumor mutation burden between subgroups

After combining the copy number dataset with the somatic mutation dataset of TCGA, we visualized the top 15 genes with the highest mutational frequencies and compared their somatic mutation status across subgroups using the R package “maftool” (Mayakonda et al., 2018). The TMB value of each patient was also calculated through “maftool”, and the Mann-Whitney or unpaired *t*-test was used to compare TMB values across subgroups (Mayakonda et al., 2018). *p*-values < 0.05 were considered statistically significant.

### Comprehensive immune characteristics analysis between subgroups

By relating gene expression data to cell purity data, the “ESITMATE” R package was utilized to determine the activities of tumor cells, immune cells, and stromal cells inside the tumor environment (Yoshihara et al., 2013). We next used single-sample GSEA through the “GSVA” R package to determine the relative proportions of 28 different types of tumor-infiltrating immune cells (Hanzelmann et al., 2013). Supplementary Table S2 contains all the gene sets for targeted immune cells. Apart from that, the relative expression levels of the ICIs-targeted genes were determined using FPKM values and compared using Mann-Whitney or unpaired *t*-test.

### Immunotherapy and chemotherapeutic response prediction

The TIDE (Tumor Immune Dysfunction and Exclusion) web tool (http://tide.dfci.harvard.edu/) was used to predict immunotherapy responses (Jiang et al., 2018). Patients with a lower TIDE score were considered to have a better response to immunotherapy. Besides, based on the GDSC (Genomics of Drug Sensitivity in Cancer) database, the R package “oncoPredict” was used to perform ridge regression analysis on each sample to predict IC50 values for targeted drugs (Maeser et al., 2021). A Mann-Whitney or unpaired *t*-test was used to compare TIDE scores and IC50 values across subgroups. *p*-values<0.05 were considered statistically significant.

## Results

### Alterations of pyroptosis-related genes RNA expression in pancreatic cancer

**FIGURE 2:**
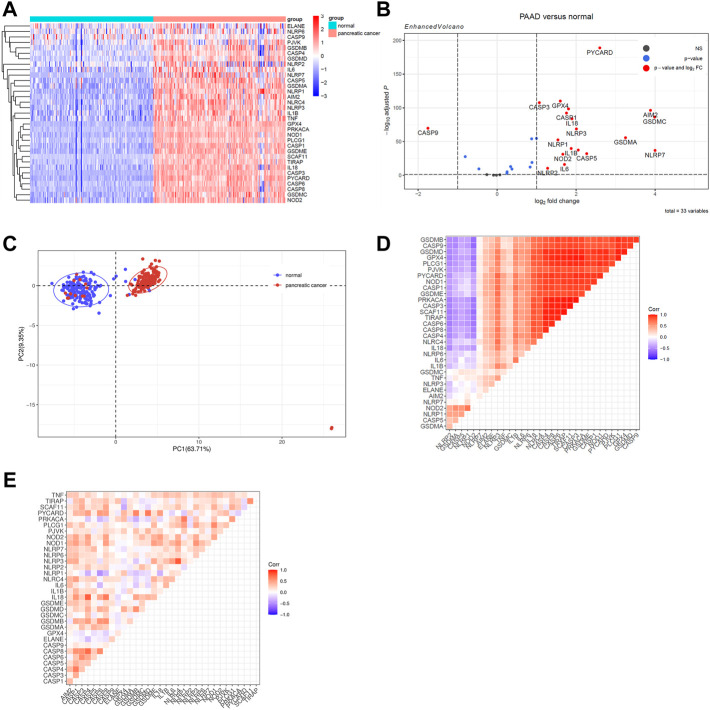
Identify differentially expressed PRGs between PAAD and normal pancreatic tissue. Genes with |log2 fold change (log2FC) | > 1 and adjusted *p* value < 0.05 were considered as differentially expressed genes. **(A)**A heatmap to show PRGs expression within normal tissue (FPKM data from GTEx cohort) and PAAD tissue (FPKM data from TCGA cohort). **(B)** The Volcano plot created using the “Enhanced Volcano” R package to show differently expressed PRGs. **(C)** Principal component analysis was processed to identify PRGs expression characters between the normal pancreatic tissue and the PAAD tissue. **(D)** A heatmap of correlation matrix of the PRGs within normal tissue from GTEx cohort. **(E)** A heatmap of correlation matrix of the PRGs within PAAD tissue from TCGA cohort.

To begin, we identified differentially expressed PRGs between PAAD tissue and normal pancreatic tissue from the TCGA-GTEx integrated cohort. The heatmap of PRGs revealed that nearly all PRGs are significantly overexpressed within PAAD tissue (Figure 2A). More specifically, the expression of AIM2, CASP1, CASP3, CASP5, GSDMA, GSDMC, IL1B, IL6, IL18, NLRP1, NLRP2, NLRP3, NLRP7, NOD2, TNF, GPX4, and PYCARD increased more than twofold, whereas CASP9 expression decreased (Figure 2B). Following that, we analyzed two additional GEO datasets (GSE28735 and GSE62452) to see whether this differential expression is widespread, which showed a significantly less trend of increase (Supplementary Figures S1A,B) (Zhang et al., 2012; Yang et al., 2016). Considering that the samples of GSE28735 and GSE62452 were taken from tumor and paired adjacent normal tissue, while control samples for the TCGA cohort were derived from healthy pancreas samples from a different cohort, the batch effects may partially account for the difference. Nevertheless, all three cohorts revealed unequivocally that PRGs were activated in PAAD and 18 of these PRGs were overexpressed in all of the datasets when setting *p* < 0.05 as threshold. We next enriched these 18 PRGs into pyroptosis signaling pathways and discovered that caspase-1, 3, and 8-dependent pyroptosis, as well as gasdmin B-mediated pyroptosis, were all closely related with pancreatic cancer (Supplementary Figure S1C). In general, multiple pyroptosis mechanisms are commonly activated in pancreatic cancer.

Then, principal component analysis was processed to identify PRGs expression characteristics between normal pancreatic tissue and PAAD, which revealed a clear distinction among samples (Figure 2C). To achieve a better understanding of the relationship among PRGs, the correlation matrix was constructed by calculating the Pearson correlation coefficient between each two genes within either normal samples from the GTEx cohort or PAAD samples from the TCGA cohort. In normal pancreatic tissue, the majority of PRGs were found to be remarkably positively linked with each others while only five genes were shown to be adversely connected to other PRGs, including NLRP2, GSDMA, CASP5, NLRP1, and NOD2 (Figure 2D). Among the PAAD samples, the expression of PRGs was likewise positively correlated, which suggested that the co-interaction of PRGs may have a role in PAAD development (Figure 2E).

### DNA methylation and copy number variation affect the pyroptosis-related genes expression

**FIGURE 3:**
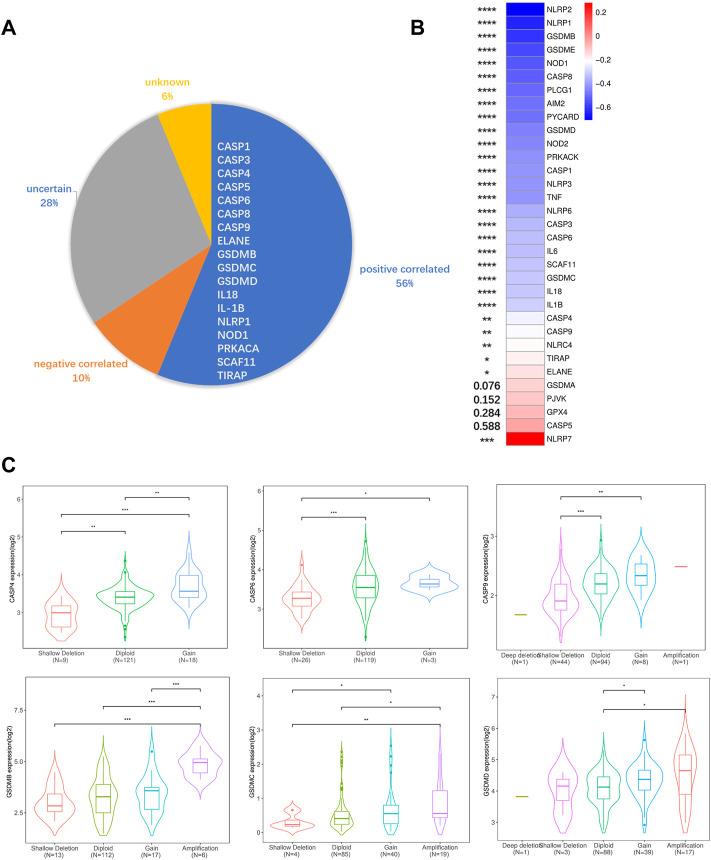
DNA methylation, CNV, and gene expression correlation analysis. **(A)** Correlations between CNV and PRGs expression. Positive correlation was defined as certain PRGs expression increased while copy number augmented. Negative correlation was defined as certain PRGs expression decreased while copy number augmented. Uncertain was defined as both expression increasement and decrement can be observed while copy number augmented. Unknown was defined as no significant differences between different CNV groups. **(B)** Pearson correlative value between methylation (HM450) versus mRNA expression z-scores relative to all samples of each PRGs. **(C)** Violin plots of example positive correlated PRGs. The rest PRGs are presented in Supplementary Figure S2. Significance was determined using the Mann-Whitney or unpaired t-test. Data shown are means ± SD, *p < 0.05, **p < 0.01, ***p < 0.001, ****p < 0.0001.

To elucidate possible explanations for the increased expression of PRGs in the TCGA cohort, we analyzed DNA methylation and CNV. Both DNA methylation and CNV have been implicated in the regulation of gene expression in a variety of cancers (Stranger et al., 2007; Daniel et al., 2011). To ascertain if CNV influences PRGs expression, we divided the TCGA cohort into five or fewer groups based on their copy number for each gene, which included deletion, shallow deletion, diploid, gain, and amplification. We discovered that copy number is positively correlated with gene expression in more than half of the PRGs, suggesting a significant role for CNV in gene regulation. Besides that, copy number is negatively correlated with gene expression in 10% of PRGs and has no correlation in the remaining PRGs. (Figures 3A,C; Supplementary Figure S2). Since the CNV alone could not fully account for the increased PRGs expression, we performed a correlation analysis between DNA methylation and PRGs expression, revealing that the expression of 28/33 PRGs is negatively correlated with DNA methylation (Figure 3B). This indicates both DNA demethylation and copy number increasement contribute to the overexpression of PRGs in PAAD.

### Construction of a prognostic gene signature

**FIGURE 4:**
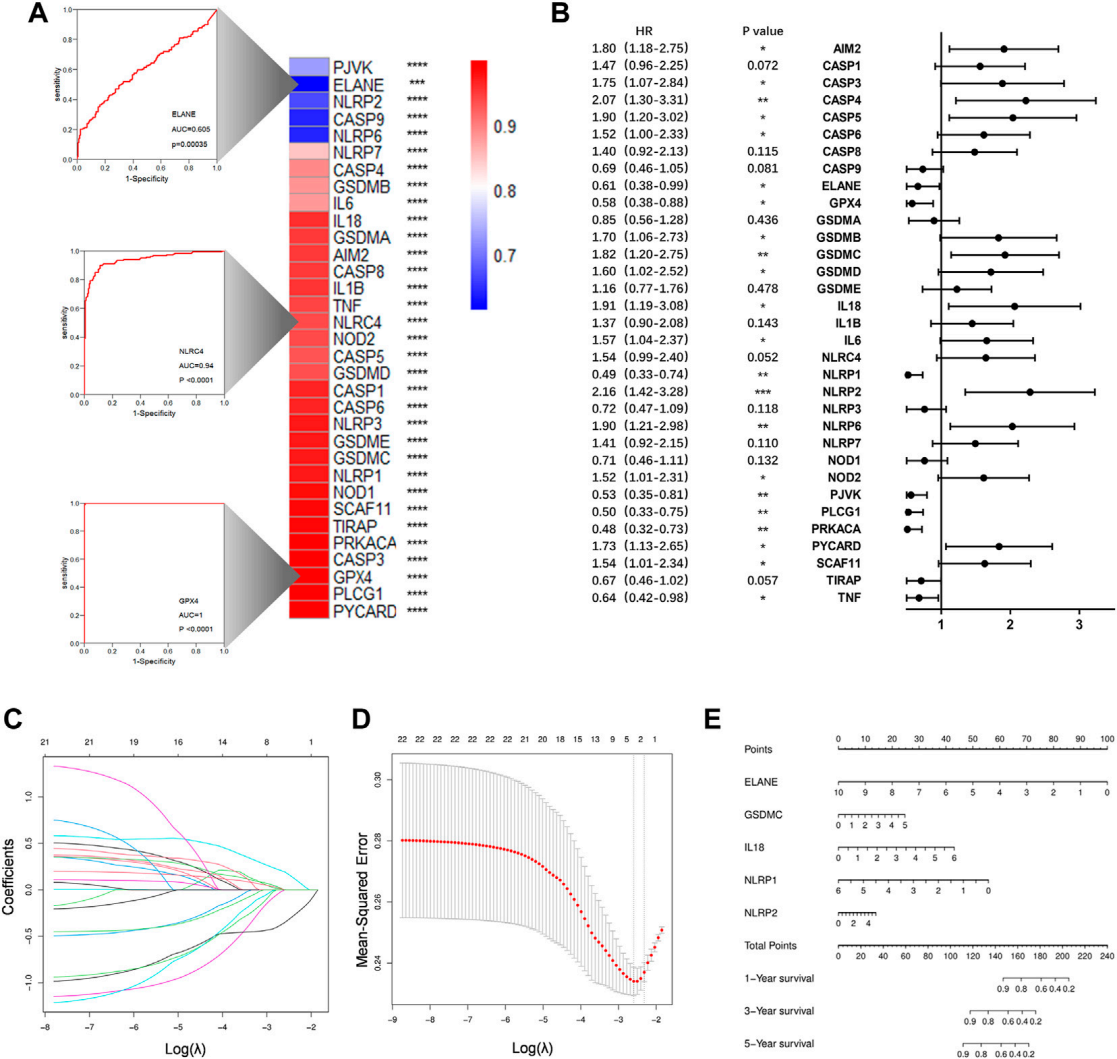
Construction of a prognostic prediction model. **(A)**Heatmap to show AUC values for each PRGs. Three example ROC curves are displayed on the left. **(B)** Hazard ratios analyzed *via* univariate cox regression to evaluate the prognostic ability for each PRGs. **(C)** LASSO coefficient profile of PRGs. **(D)** Ten times cross-validation for parameter selections in the LASSO cox regression. **(E)**The nomogram incorporating 5 selected PRGs. *p < 0.05, **p < 0.01, ***p < 0.001, ****p < 0.0001.

The ROC curves for each PRGs revealed that the majority of PRGs had a high predictive value for diagnosis, implying that they may contribute to PAAD tumorigenesis (Figure 4A). To further assess their prognostic potential, we performed a univariate cox analysis between each PRG and OS, and 22 genes were screened out (with *p* < 0.05) (Figure 4B). Lasso regression analysis was then used to identify the most prognostic genes, and 5 genes were chosen by the vertical grey line in Figure 4D (Figures 4C,D). Finally, the model was determined by multivariate cox regression within selected PRGs. Among them, GSDMC, IL18, and NLRP2 are all associated with an increased risk, while the other two confer a protective effect (Figure 4E). The formula of the risk score was: risk score = (GSDMC*0.2302) -(ELANE*0.4664)+ (IL18*0.3341)—(NLRP1*0.4324)+ (NLRP2*0.1297). Taking the median risk score as the cut-off value, we classified all TCGA patients into low- and high-risk groups. Detailed clinical information is presented in Table 1 Regardless of histologic stage, disease type or OS, the majority of clinicopathological characteristics are evenly distributed among two groups. An increased risk score, on the other hand, may indicate a higher histological grade and a greater likelihood of ductal and lobular origins.

**TABLE 1:** Clinical characteristics between risk score related subgroups.

	Total (*n* = 177)	Risk level		*p*-value
		low (*n* = 88)	High (*n* = 89)	
Age(year)				0.8259
<65	81 (45.76%)	41 (46.59%)	40 (44.94%)	
≥65	96 (54.24%)	47 (53.41%)	49 (55.06%)	
Gender				0.4021
Male	97 (54.80%)	51 (57.95%)	46 (51.69%)	
Female	80 (45.20%)	37 (42.05%)	43 (48.31%)	
TNM stage				0.2225
Stage I	21 (11.86%)	14 (15.91%)	7 (7.87%)	
Stage II	145 (81.92)	70 (79.55.22)	75 (84.27%)	
Stage III-IV	8 (4.52%)	3 (3.41%)	5 (5.62%)	
Unknown	3 (1.69%)	1 (1.14%)	2 (2.25%)	
Histologic grade				0.0085
G1-G2	125 (70.62%)	70 (79.55%)	55 (61.80%)	
G3-G4	50 (28.25%)	17 (19.32%)	33 (37.08%)	
unknown	2 (1.13%)	1 (1.14%)	1 (1.12%)	
Disease type				0.0553
Adenomas and adenocarcinomas	30 (16.95%)	21 (23.86%)	9 (10.11%)	
Cystic, mucinous, and serous neoplasms	5 (2.82%)	3 (3.41%)	2 (2.25%)	
Ductal and lobular neoplasms	141 (79.66%)	63 (71.59%)	78 (87.64%)	
Epithelial neoplasms, NOS	1 (0.56%)	1 (1.14%)	0	
Family history of cancer				0.5449
YES	62 (35.03%)	32 (36.36%)	30 (33.71%)	
NO	47 (26.55%)	27 (30.68%)	20 (22.47%)	
Unknown	68 (38.42%)	29 (32.95%)	39 (43.82%)	
Family history of pancreatitis				0.7299
Yes	13 (7.34%)	6 (6.82%)	7 (7.87%)	
No	127 (71.75)	65 (73.86%)	62 (69.66%)	
Unknown	37 (20.90%)	17 (19.32%)	20 (22.47%)	
Overall survive				<0.0001
Alive	85 (48.02%)	59 (67.05%)	26 (29.21%)	
Dead	92 (51.98%)	29 (32.95%)	63 (70.79%)	

### Prognostic value of pyroptosis-related genes signature in TCGA and validation cohort

**FIGURE 5:**
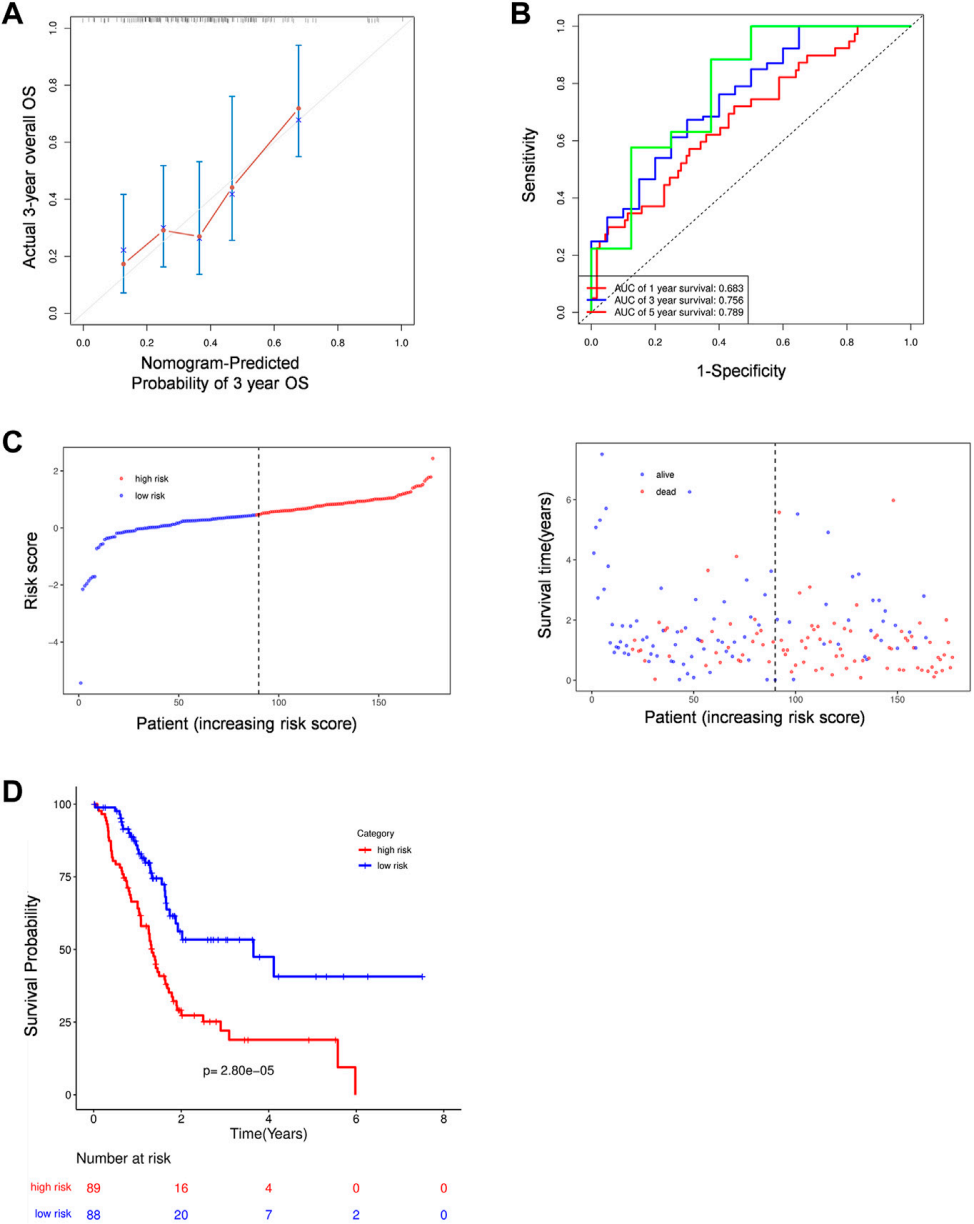
The prognostic analysis of PRGs signature. **(A)** Calibration plots of the nomogram for predicting OS within 3 years basing on PRGs signature in the TCGA cohorts. **(B)** Time dependent ROC analysis in the TCGA cohort. **(C–D)** The plots of risk score and alive status**(C)** as well as Kaplan-Meier survival analysis **(D)** in the TCGA cohorts.

To assess the prognostic efficacy of this signature, we calculated the probability of 3-years OS in the TCGA cohort (Figure 5A) and a validation cohort, ICGC (Supplementary Figure S3A). The results indicated that the model had a high predictive capacity in both cohorts. Additionally, time dependent ROC analysis was used to assess the sensitivity and specificity of this model. As for the TCGA cohort, beside 1-year, both 3-years and 5-years corresponding areas under the curve (AUC) are over 0.75 (Figure 5B), whereas the ICGC cohort’s accuracy is lower, with a 1-year AUC of 0.661 and a 3-years AUC of 0.528 (Supplementary Figure S3B). However, its poor performance for predicting longer time survival status may be explained by the fact that only 10% of patients in the ICGC cohort survive till the third year. Following that, similar to the TCGA cohort, all 89 patients in the ICGC cohort were equally divided into low- and high-risk groups based on their risk score, and we observed an obvious difference in OS between the two groups. Higher risk patients were associated with more deaths and tended to have shorter survival time in both cohorts (Figures 5C,D; Supplementary Figures S3C,D).

### pyroptosis-related genes model outperforms clinical characteristics in prognosis

**FIGURE 6:**
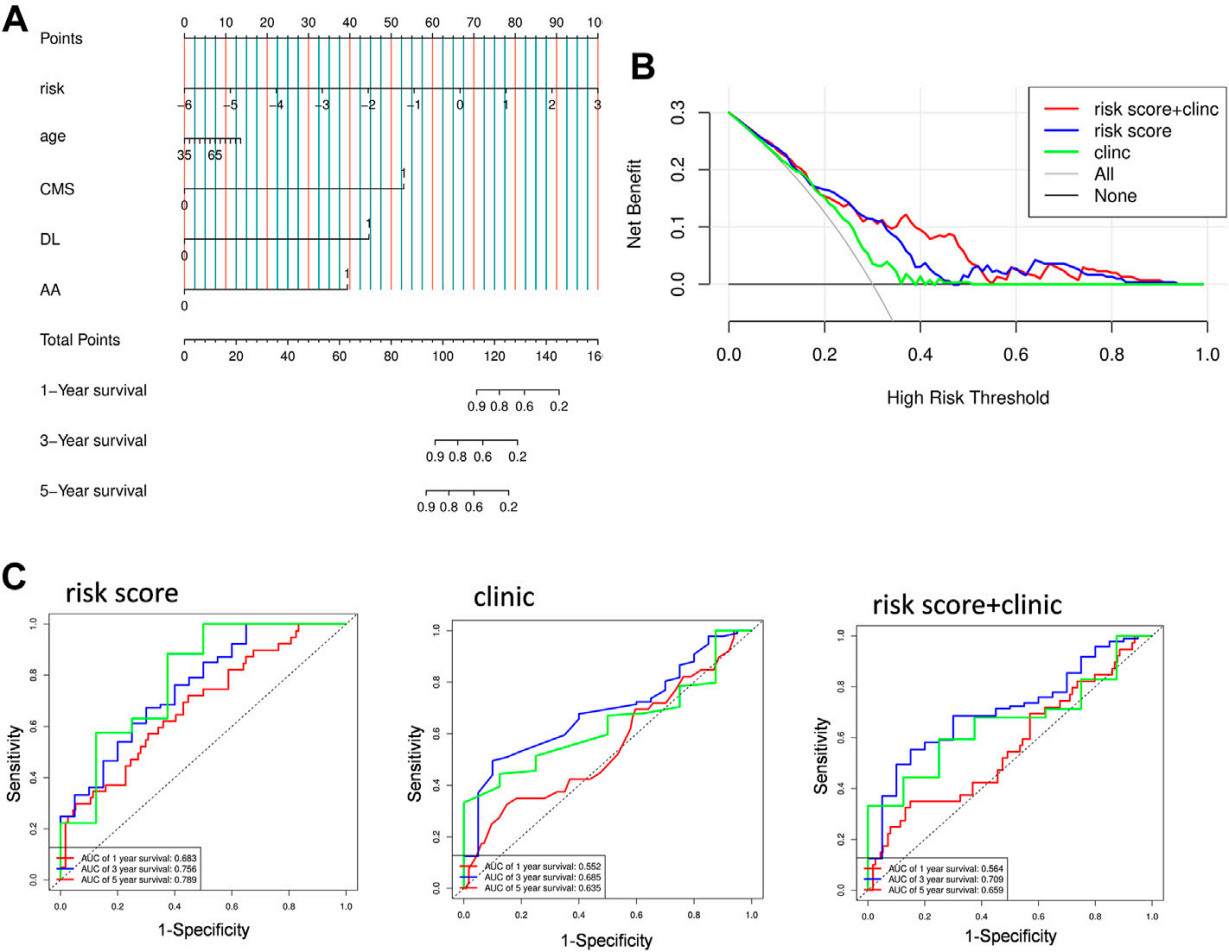
Validation prognostic efficiency of PRGs signature. **(A)** Nomogram predicted 1- ,3-, and 5-years OS based on prognostic model combined with clinical characteristic in the TCGA cohort. CMS: Cystic, Mucinous and Serous Neoplasms; DL: Ductal and Lobular Neoplasms; AA: Adenomas and Adenocarcinomas. **(B)** The decision curve of the risk score, clinical characteristic and their combination. **(C)** time-dependent ROC curves for the risk score, clinical characteristic, or their combination.

Following that, we compare the predictive accuracy of our model to that of clinicopathological characteristics. Both univariate and multivariate analyses indicated that risk score is an independent predictor, moreover, age and disease type also demonstrated their independent predictive ability with *p* < 0.1 as the threshold value (Table 2). After combining these three variables, a nomogram model was built to evaluate its clinical utility (Figure 6A). Then, we processed decision curve and ROC analysis to compare the clinical benefit of the composite nomogram to that of a risk score or clinical characteristics alone. While the composite model performed better than the basic clinical factors in terms of prognosis accuracy, it demonstrated limited clinical net benefit compared to the risk score (Figure 6B). Additionally, the time-related AUCs of the risk score model were consistently greater than those of the composed model at each time point, suggesting that the risk score possessed the greatest clinical utility (Figure 6C).

**TABLE 2:** Univariate and multivariate cox regression analysis for prognostic model and clinical characteristics.

Variable	Univariate analysiss		Multivariate analysiss	
	Hazard ratio (95% Cl)	*p*-value	Hazard ratio (95% Cl)	*p*-value
Risk score	2.72 (1.88–3.93)	<0.0001	2.52 (1.69–3.76)	<0.0001
Age	1.03 (1.01–1.05)	0.0076	1.02 (1.00–1.04)	0.0559
Gender				
Female	References			
Male	0.81 (0.54–1.22)	0.3111		
Tumor stage				
Stage I	References			
Stage II	2.33 (1.07–5.09)	0.0334		
Stage III	1.25 (0.15–10.28)	0.8323		
Stage IV	1.56 (0.32–7.61)	0.5824		
Histology grade				
G1	References			
G2	1.95 (1.00–3.79)	0.0487		
G3	2.62 (1.30–5.27)	0.0071		
G4	1.65 (0.21–12.85)	0.6346		
Disease type				
AA^a^	References		References	
CMS^a^	4.80 (1.27–18.21)	0.0210	3.24 (0.82–12.86)	0.0948
DL^a^	3.16 (1.52–6.57)	0.0020	1.52 (0.71–3.25)	0.2786
History of chronic pancreatitis				
No	References			
Yes	1.18 (0.56–2.47)	0.6649		
Family history of cancers				
No	References			
Yes	1.12 (0.65–1.92)	0.6858		

a AA, is short for Adenomas and Adenocarcinomas.

CMS, is short for Cystic, Mucinous and Serous Neoplasms.

DL, is short for Ductal and Lobular Neoplasms.

### Bioinformation analysis based on the pyroptosis-related genes model

**FIGURE 7:**
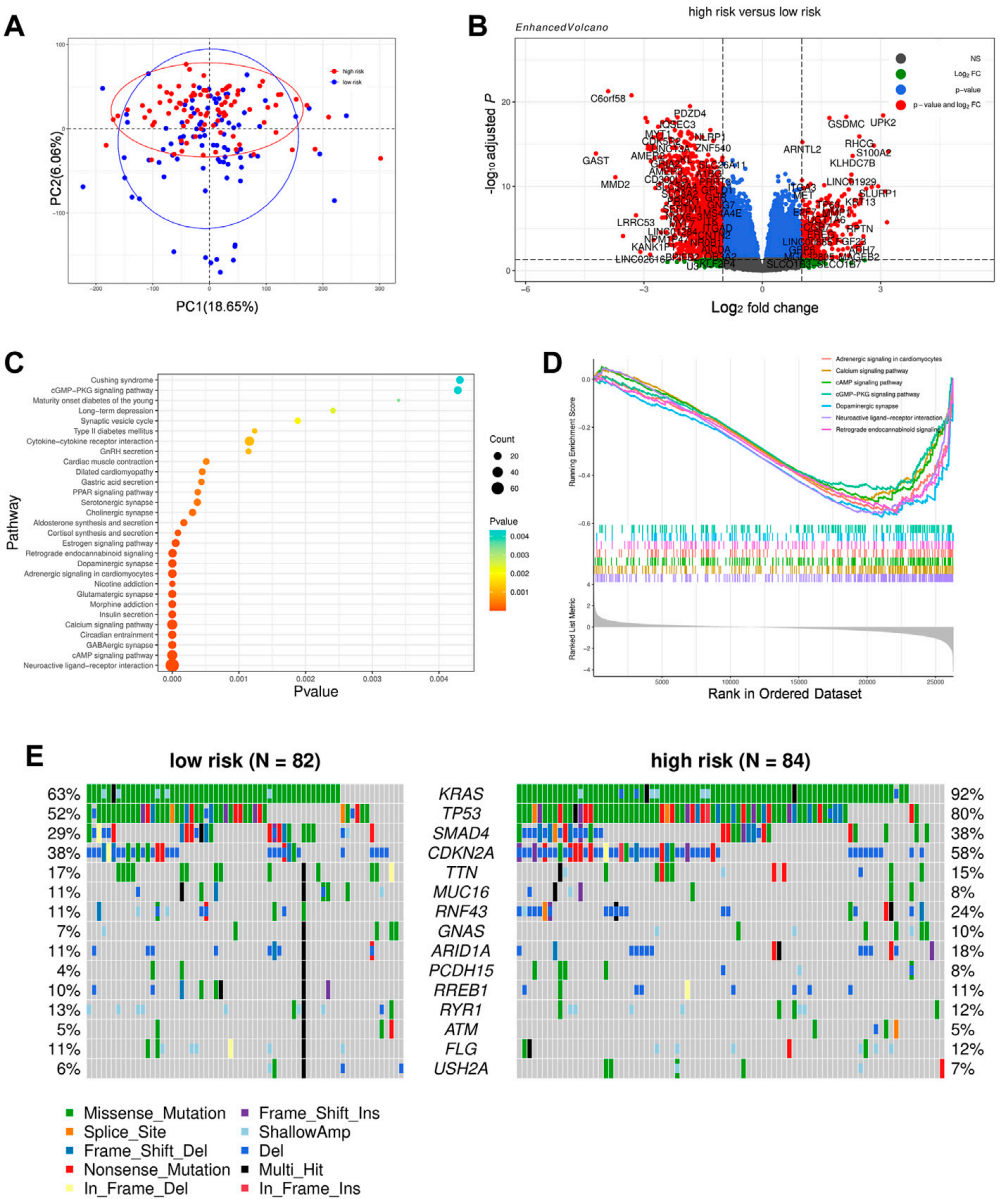
Comparison of the subgroups of TCGA cohort. **(A)** Principal component analysis of the TCGA cohort grouped by high and low risk. **(B)** A volcano plot represented DEGs between the high- and low-risk groups of TCGA cohort. **(C)** Function enrichment analysis of DEGs based on the KEGG signaling pathway. **(D)**GSEA result of DEGs based upon KEGG signaling pathway. **(E)** Distribution of frequently mutated genes in different TCGA subgroups.

We identified 365 genes with increased expression and 1,514 genes with decreased expression in the high-risk group as compared to the low-risk group (Figures 7A,B). These DEGs were then used to conduct KEGG enrichment and GSEA analysis to further investigate the biological pathway correlated with risk score. Interestingly, DEGs were predominantly enriched in organismal systems such as endocrine, nervous, and circulatory systems (Figure 7C). Meanwhile, the GSEA results demonstrate that several pathways, including calcium signaling, cAMP signaling, cGMP-PKG signaling pathways and so on, are down-regulated in the high-risk group (Figure 7D). Apart from functional analysis, we then looked at the somatic mutation status of TCGA patients. As expected, high-risk individuals have a considerably higher somatic mutation burden, typically for the genes KARS and TP53, which are known to be the primary drivers of PAAD (Kleeff et al., 2016) (Figure 7E; Supplementary Figure S4A). Consistently, the tumor mutation burden (TMB) was also found to be considerably greater in the high-risk group than in the low-risk group (Supplementary Figure S4B).

Given that KRAS and TP54 have been linked to other cell death processes such apoptosis and ferroptosis, we attempted to identity the specific correlation between oncogenes and pyroptosis by comparing the expression of PRGs between KRAS or TP53 mutated and unmutated individuals (Chen et al., 2021). Despite the fact that GSDMC, NOD2, and IL18 were modestly elevated while NLRP1 and NLRP6 were lowered, the majority of PRGs between the mutant and non-mutant groups were not significantly different (data not shown). The link between pyroptosis and gene mutation is not evident based on the existing findings, and more research is needed to understand the particular interaction between the two.

### Immunity features underlying the pyroptosis-related genes model

**FIGURE 8:**
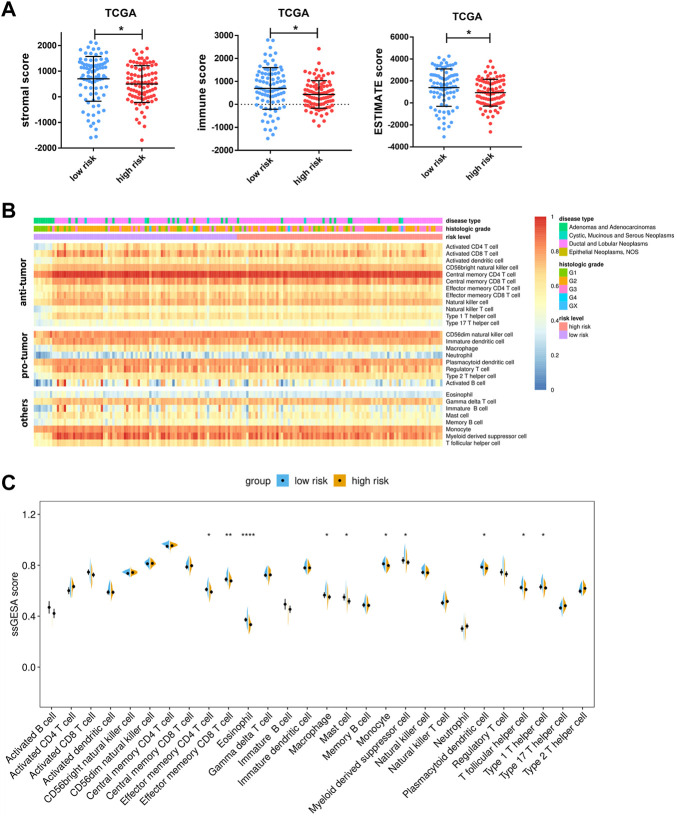
Associations between risk score and tumor microenvironment. **(A)** Comparison of stromal scores, immune scores, and ESTIMATE scores between the high- and low-risk groups of TCGA cohorts. **(B)** Heatmap of ssGSEA enrichment scores of 28 immune cell types in the TCGA cohort. Notably, the cells are grouped according to their widely accepted role in cancer, including anti-tumor, pro-tumor, and others. **(C)** Comparison of ssGSEA enrichment scores of 28 types of immune cells between the high- and low-risk groups in the TCGA cohort. Data are presented as means ± SD. Significant was determined using Mann-Whitney or unpaired *t*-test. *p < 0.05 **p < 0.005, and ****p < 0.00005.

We further characterize their immune environment heterogeneity by elucidating the association between risk score and immune state. The ESTIMATE web tool was first used to determine cell distribution, and it revealed that high-risk group had significantly less stromal cell and immune cell infiltration. Meanwhile, the testing group ICGC cohort presented a similar trend, though without a statistically significant difference (Figure 8A; Supplementary Figure S4C). Additionally, the compositions of specific cell types were determined through ssGSEA, showing that the infiltration of a considerable number of immune cell types were reduced in high risk group, including effector memory CD4+T-cells, effector memory CD8+T-cells, and type I helper cells, which are known to have anti-tumor effects. Apart from these, eosinophils, macrophages, mast cells, monocytes, myeloid derived suppressor cells, and plasmacytoid dendritic cells were found to be adversely associated with risk score (Figures 8B,C).

### Therapy response features underlying the pyroptosis-related gene model

**FIGURE 9:**
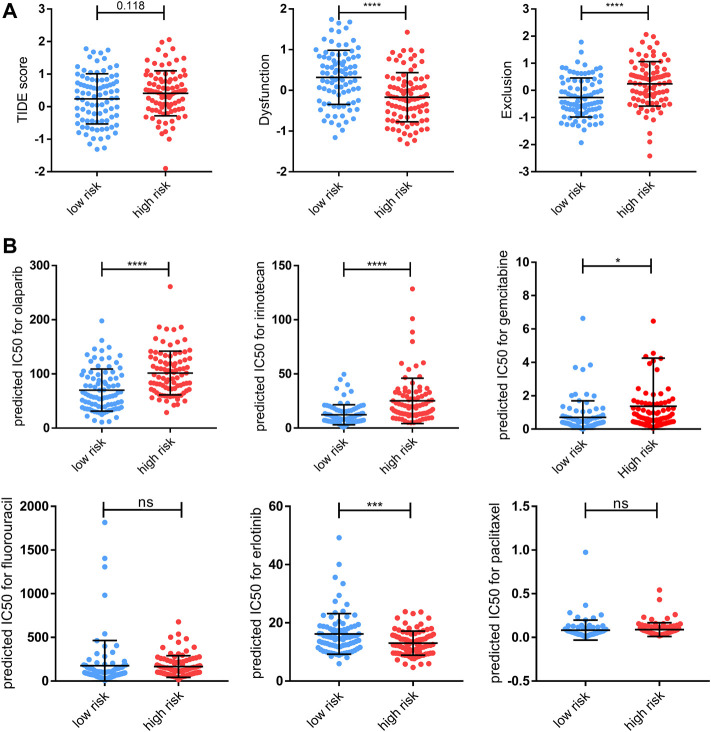
Therapy response features underlying the PRGs model. **(A)** Comparison of TIDE score, T-cell dysfunction (“Dysfunction”) score, and T-cell exclusion (“Exclusion”) scores between the high- and low-risk groups of the TCGA cohort. **(B)** Predicted IC50 for olaparib, irinotecan, gemcitabine, fluorouracil, erlotinib, and paclitaxel for low-risk and high-risk groups. Data shown are means ± SD. Symbols represent the individual patients. Significant was determined using the Mann-Whitney or unpaired *t*-test. *p < 0.05 **p < 0.005, ***p < 0.0005, and ****p < 0.00005.

We suspected that a higher risk score would be correlated with a weaker response to immunotherapy and other bio-agents, given that patients in the high-risk group exhibited reduced immune cell infiltration. Then, the TIDE analysis corroborated our hypothesis, demonstrating that individuals at low-risk are more likely to respond to ICI treatment but without statistical significance (Figure 9A). Moreover, patients in the high-risk group have higher exclusion score but a lower dysfunction score, suggesting that immunological exclusion was the primary cause of their poor outcomes (Figure 9A). Notably, while both increased and decreased expression of the ICI target gene can be observed, the link between specific ICI and risk score requires further investigation (Supplementary Figure S4D). Apart from that, we used onco predict to predict the IC50 values for FDA-approved drugs in high- and low- risk patients. Among the six most commonly used drugs, the low-risk group had considerably lower projected IC50 values for olaparib, irirntecan, and gemcitabine, implying that lower risk is associated with better outcomes from these chemotherapeutic drugs (Figure 9B). Overall, patients in the high-risk group were less sensitive to both immunotherapy and chemotherapy in general, which may have contributed to their poor prognosis.

## Discussion

PAAD is always diagnosed at an advanced stage because of the lack of identifiable symptoms, and only a minority of patients can benefit from conventional surgical treatment or cytotoxic chemotherapy (Von Hoff et al., 2013; Walter et al., 2016). As a result, PAAD is currently one of the top 10 most lethal tumors (Rahib et al., 2014). The immunosuppressive and desmoplastic milieu of PAAD is a substantial impediment to optimizing therapeutic efficacy, including difficulties in drug transport and limited responses to ICI-based immunotherapy (Li et al., 2020). Stimulating the immunogenic cell death of tumor cells is regarded to be an efficient method of converting the “cool” tumor microenvironment to a “hot” environment (Kroemer et al., 2013). Given that tumor cells show intrinsic resistance to apoptosis, targeting pyroptosis might be a more efficient strategy for boosting immunotherapy (Huang et al., 2018). Our study investigated the combined effects of various PRGs in PAAD and developed a prognostic model capable of reliably predicting patient survival status and response to prospective targeted therapy.

In this study, we were surprised to find that the majority of PRGs expressed significantly differently between normal pancreatic tissue and PAAD, reflecting a fundamental change in pyroptosis activity. Gene overexpression can occur for a variety of reasons, including gene amplification, activating mutation, or epigenetic modification (Stranger et al., 2007; Daniel et al., 2011). In our case, most of these upregulations occur in part as a result of increased copy number or demethylation. Additionally, the majority of overexpressed PRGs are strongly associated with poor prognosis, indicating that they may contribute to survival state prediction. Thus, using univariate cox and lasso regression to avoid overfitting, five prognostic PRGs were chosen. Following that, we generated a signature comprised of five PRGs (ELANE, GSDMC, IL18, NLRP1, and NLRP2) by multivariate cox, which named risk score, and validated its accuracy in both the training and validation cohorts. Among these core genes, higher ELANE and NLRP1 expression suggested a favorable prognosis for the patients. Consistently, Cui et al. (2021) recently demonstrated that neutrophil-derived active neutrophil elastase (ELANE) not only kills numerous types of cancer cells while sparing proximal non-cancer cells by liberating the CD95 death domain that interacts with histone H1 isoforms, but also inhibits metastasis *via* CD8+T mediated abscopal effect. Furthermore, it has been discovered that NLRP1 downregulation promotes tumorigenesis, including lung adenocarcinoma and colorectal cancer (Chen et al., 2015; Shen et al., 2021). On the other hand, overexpression of GSDMC, IL18, and NLRP2 were associated with a poor prognosis in patients with PAAD. Hou et al. (2020) showed GSDMC mediated non-canonical pyroptosis upon caspase-8 activation and that high GSDMC expression correlated with poor survival. It is difficult to thoroughly elucidate the role of IL18 in cancer. A high level of IL18 in pancreatic tumor tissue was associated with a shorter survival time, increased invasion, and metastasis, whereas a high IL18 level in plasma was correlated with a longer survival time (Guo et al., 2016). By combining our signature with previous studies, we were able to confirm and truly illustrate the predictive usefulness of these core PRGs.

Additionally, the singnature revealed differences in several pathways between the two groups. Due to the fact that the number of downregulated genes was much more than the number of upregulated genes, the majority of pathways, such as GABAergic synapase and insulin secretion, were enriched by downregulated genes, and these pathways may have a correlation with PAAD progression and prognosis. For example, gaba suppresses PAAD by inhibiting the β-adrenergic cascade and nicotine-induced cell proliferation (Al-Wadei et al., 2011; Al-Wadei et al., 2013; Al-Wadei et al., 2016). cAMP has both pro- and anti-tumor effects in malignancies (Tagliaferri et al., 1988; Ligumsky et al., 2012; Almahariq et al., 2015); To our surprise, the calcium signaling pathway and the neuroactive ligand-receptor interaction pathway, both of which are associated with a poor prognosis (Bettaieb et al., 2021; Qian et al., 2021), were downregulated in the high-risk group. However, the link between pyroptosis and these pathways is currently unknown and needs further investigation.

The pro- or anti- tumor effects of proptosis are somehow determined by the surrounding microenvironment (Hou et al., 2021). Several investigators reported the pyroptosis of tumor cells can induce inflammatory response in microenvironment and attracting CD4^+^ and CD8+T-cell populations (Wang et al., 2020). In our case, though multiple PRGs are robustly overexpressed within PAAD, it is evident pancreatic tumor microenvironment exhibits an immunosuppressive condition (Zhu et al., 2014; Jiang et al., 2016; Kumar et al., 2022). One possible explanation for this is that, unlike acute pyroptosis induction, chronic induction of pyroptosis in some tumors can result chronic inflammation, which leads to a tumor-promoting microenvironment (Tsuchiya, 2021). Besides, extracellular ATP released from pyroptotic cells can be rapidly broken down into adenosine, an immunosuppressive substance, the gradual release of modest amounts of ATP from pyroptotic tumor cells may impact antitumor immunity (Vultaggio-Poma et al., 2020; Tsuchiya, 2021). Apart from that, the pytoptosis that happened in the center region of the tumor could result in chronic tumor necrosis, which suppressed the anti-tumor immunity and accelerated tumor progression (Hou et al., 2020). In our model, patients with lower risk scores were infiltrated with more immune cells, including several anti-tumor immune cells. So that if therapy-induced pyroptosis is expected to improve the pancreatic tumor microenvironment it may be important to determine the appropriate extent of pyroptosis induction, which should be neither too strong nor too weak (Tsuchiya, 2021).

Apart from the immune cell landscape, this signature also showed a significant correlation with somatic mutation status and therapeutic response. The patients with higher risk scores carried more mutation burden, with more mutations in KARS, TP53, ADAMTS12, SMAD4 FAT4, DCHS1, and CDKN2A mutations. Among these genes, KARS, CDKN2A, TP53, and SMAD4 are four major genes involved in the progression of PAAD (Kleeff et al., 2016). However, it is unclear whether these oncogenes are involved in pyroptosis. Moreover, TIDE analysis revealed that PAAD patients with lower risk scores had a higher likelihood of achieving durable benefits from immunotherapy. PAAD is also characterized by a remarkable tolerance to chemotherapy (Kleeff et al., 2016). Thus, to test the PRGs signature’s predictive utility in clinical practice, we next predicted the sensitivity to FDA-proved PAAD chemotherapeutic drugs based on gene expression profiles. Similar to immunotherapy, a low-risk score was associated with a better response to olaparib, irinotecan, and gemcitabine. In general, our findings demonstrated that patients with low-risk scores were more likely to be have a reduced mutation burden and benefit from both immunotherapy and chemotherapy.

In this study, we created a valuable PRGs signature and thoroughly explored its correlations with prognosis, immune infiltration, somatic gene mutation, and treatment response. Our model performs well in predicting patient prognosis and treatment response. Moreover, we laid the groundwork for a more complete understanding of pyroptosis’s role in PAAD. However, our work is still in its early stage and the limitations of this study are clear. Further clinical trials need to be conducted to fully verify the accuracy of this model. The true involvement of pyroptosis in cancer remains a mystery, and additional researches are required.

## Data Availability

The original contributions presented in the study are included in the article/Supplementary Material, further inquiries can be directed to the corresponding authors.
