# Supplementary figures and images for: A pyroptosis-related gene signature for prognosis and immune microenvironment of pancreatic cancer
Source: Front Genet. 2022 Aug 29;13:817919. doi: 10.3389/fgene.2022.817919 (PMC9476319; doi:10.3389/fgene.2022.817919)

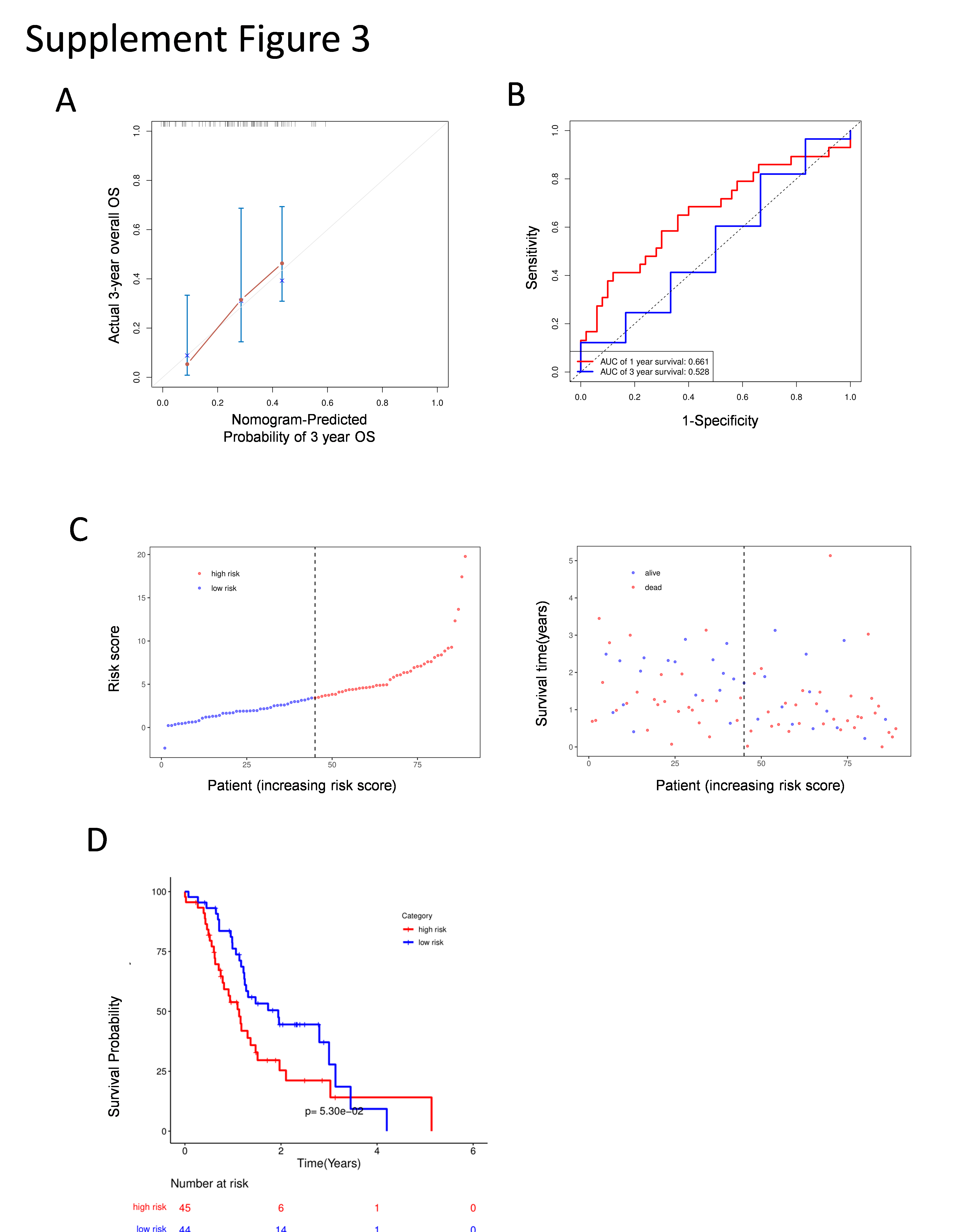

Supplement: Supplementary file 3 [file Image3.TIF]

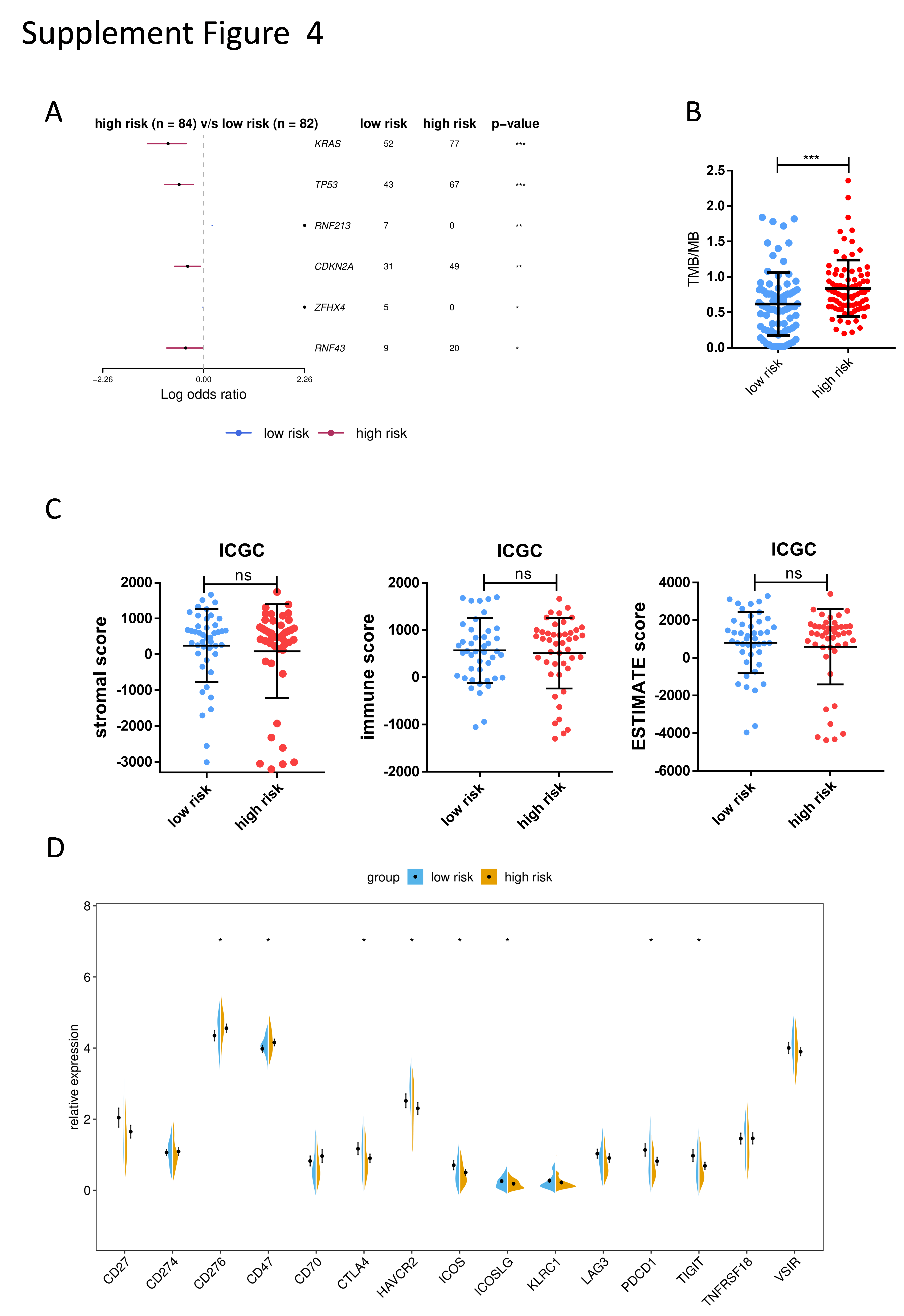

Supplement: Supplementary file 4 [file Image4.TIF]

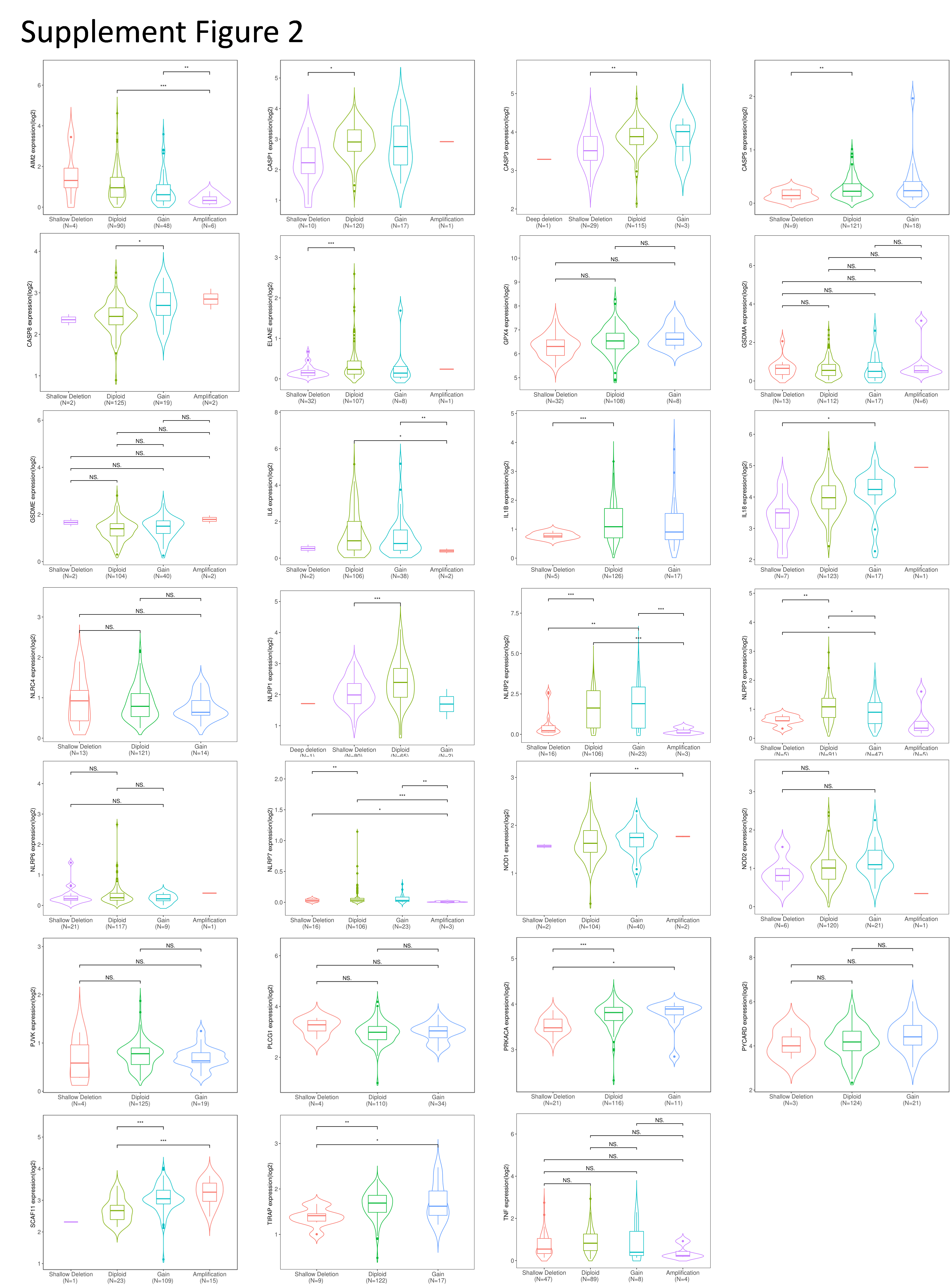

Supplement: Supplementary file 5 [file Image2.TIF]

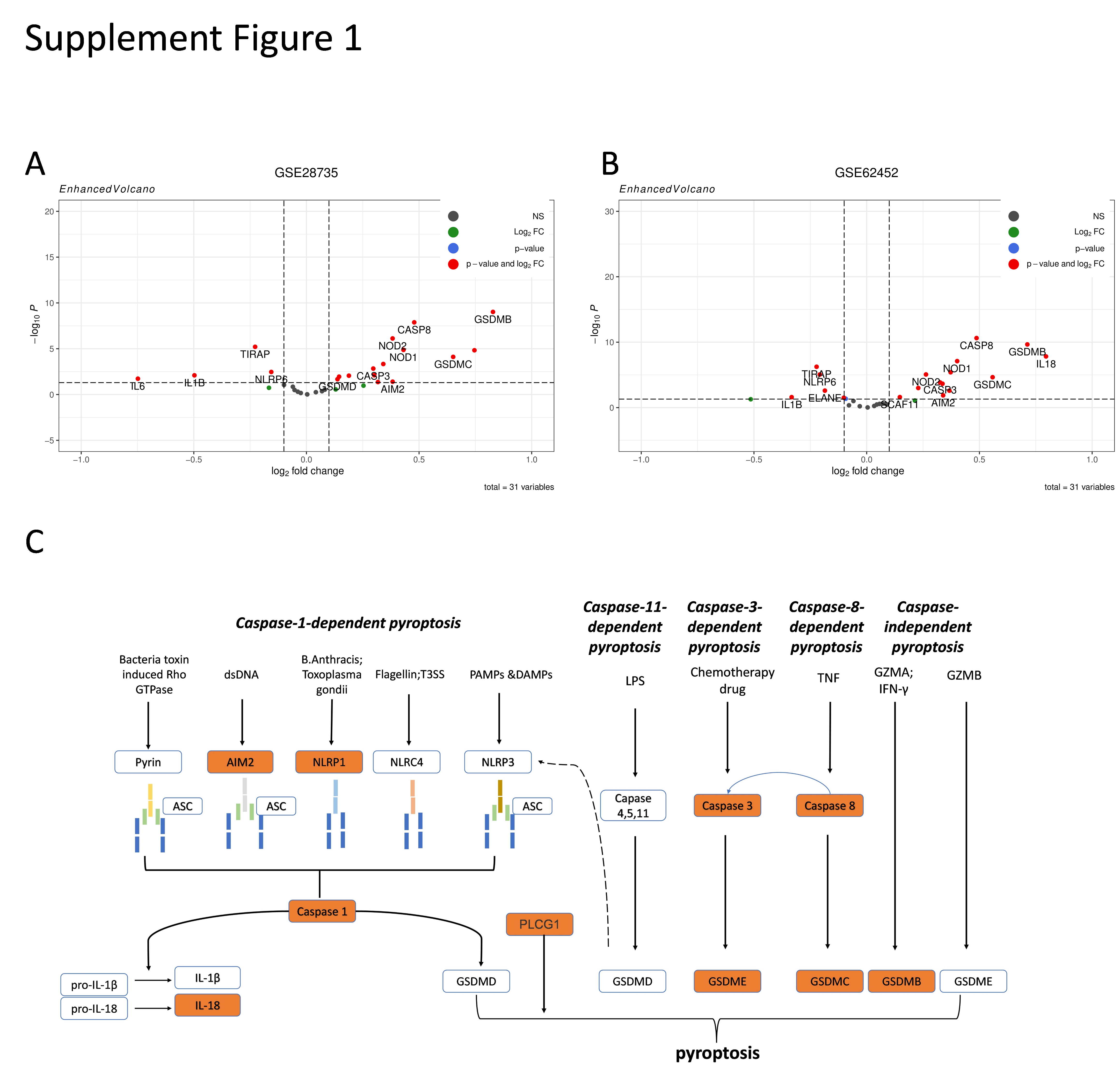

Supplement: Supplementary file 6 [file Image1.TIF]
