# Supplementary material for: A pyroptosis-related gene signature for prognosis and immune microenvironment of pancreatic cancer
Source: Front Genet. 2022 Aug 29;13:817919. doi: 10.3389/fgene.2022.817919 (PMC9476319; doi:10.3389/fgene.2022.817919)
Supplement: Supplementary file 7 [file Table3.docx]

**Supplement figure legend**

Supplement figure 1 Differentially expressed PRGs between PAAD tissue and adjacent tissue using the data from the GEO database with the access number GSE28735(A) and GSE62452(B). (C) Molecular mechanism of pyroptosis. PRGs that were overexpressed in all datasets were highlighted in orange.

Supplement figure 2 Violin plots of PRGs not depicted in figure 2. Significance was determined using the Mann-Whitney or unpaired t-test. Data are presented as means ±SD, *p < 0.05, **p < 0.01, ***p < 0.001, ****p < 0.0001.

Supplement figure 3 The prognostic analysis of PRGs signature.

(A)Calibration plots of the nomogram for predicting OS within 3 years basing on PRGs signature in the ICGC cohorts. (B)Time dependent ROC analysis in the ICGC cohort. (C-D) The plots of risk score and alive status(C) as well as Kaplan-Meier survival analysis(D) in the ICGC cohorts.

Supplement figure 4 (A) Identified and analyzed significant differentially mutant genes between subgroups using maftool. (B) Difference of TMB between subgroups. (C) Comparison of stromal scores, immune scores, and ESTIMATE scores between the high- and low-risk groups of ICGC cohorts. (D) Relative expression of ICI-related genes in the two subgroups. Significance was determined using the Mann-Whitney or unpaired t-test. Data are presented as means ±SD, ns p≥0.05, *p < 0.05, **p < 0.01, ***p < 0.001.
